# A Survey of COVID-19 Preparedness Among Hospitals in Idaho

**DOI:** 10.1017/ice.2020.218

**Published:** 2020-05-11

**Authors:** Anubhav Kanwar, Susan Heppler, Kalpana Kanwar, Christopher K. Brown

**Affiliations:** 1 Infectious Diseases, HIV and Travel Medicine, Tri-State Memorial Hospital, Clarkston, Washinton; 2 Infection Prevention, Saint Alphonsus Medical Center, Nampa, Idaho; 3 Spokane Falls Community College, Pullman, Washington; 4 Directorate of Technical Support and Emergency Management, Occupational Safety and Health Administration, US Department of Labor, Washington, DC

## Abstract

**Background:**

SARS-CoV-2 has been implicated in the largest recorded coronavirus outbreak to date. Initially, most COVID-19 cases were in China, but the virus has spread to more than 184 countries worldwide, and the United States currently has more cases than any other country.

**Objective:**

With person-to-person spread expanding in the United States, we describe hospital preparedness for managing suspected and confirmed COVID-19 patients.

**Design:**

Cross-sectional survey focused on various elements of respiratory disease preparedness.

**Setting:**

Critical access hospitals (CAHs) and acute-care hospitals (ACHs) in Idaho.

**Methods:**

The electronic survey was sent to infection preventionists (IPs) and nurse administrators in 44 hospitals in Idaho.

**Results:**

Overall, 32 (73%) hospitals responded to the survey. Participating facilities reported their preparedness with respect to existing, formalized structures for managing infectious disease incidents—specifically COVID-19—as well as availability of resources, such as isolation rooms and personal protective equipment, for safely managing suspected and confirmed COVID-19 cases.

**Conclusions:**

Hospitals covered by the survey had varying levels of preparedness for managing COVID-19 cases, with differences across the various categories of interest in this study. Although the study reveals strengths, including in application of emergency management and infection control frameworks, it also suggests that other areas, such as consistent implementation of federal guidelines and requirements for infection prevention, are potential areas for strengthening preparedness for SARS-CoV-2 and other respiratory pathogens with pandemic potential.

Since the 1960s, human coronaviruses have been recognized as a cause of typically mild respiratory illness without any reports of epidemic spread until 2002.^1^ The 2002–2003 outbreak of severe acute respiratory syndrome (SARS)-CoV-1 and clusters of Middle East Respiratory syndrome (MERS)-CoV since 2012 have changed this notion, with thousands of cases, hundreds of deaths, and significant global economic impacts attributable to SARS-CoV-1, in particular.^2–4^ Most recently, a novel coronavirus, SARS-CoV-2, has been implicated in the largest recorded coronavirus outbreak to date, causing around 3.3 million cases of 2019 coronavirus disease (COVID-19) and >240,000 deaths globally (as of May 2, 2020).^5,6^ Although most COVID-19 cases have occurred in the United States, the virus has spread to >184 countries worldwide. In the United States, there have been >1.09 million cases with >64000 deaths (as of May 2, 2020).^6^

Because of the significant airborne transmission component associated with SARS-CoV-2, the viruses spread easily between unprotected close contacts (eg, those living with or caring for infected individuals).^7^ As epidemiologic evidence associated with SARS-CoV-2 spread has shown previously and, now, in the midst of the ongoing SARS-CoV-2 outbreak, these viruses can also lead to pandemic conditions as they spread to immunologically naïve populations worldwide.

With the ongoing SARS-CoV-2 outbreak, there is also concern of transmission to healthcare workers (HCWs). Early in the outbreak, many HCWs were infected in Hubei Province, China, before experts began to understand SARS-CoV-2 transmission. Although fewer cases were identified among HCWs as the virus spread outside Hubei Province, workers may be at risk whenever training and resources for safely managing cases are insufficient. Because additional cases will continue to occur with community transmission in the United States, it is very important to assess preparedness of hospitals to manage suspected and confirmed COVID-19 cases. Although public health agencies, including the Centers for Disease Control and Prevention (CDC), have shared interim guidance on infection prevention and control in US hospitals, gaps in hospital preparedness may lead to healthcare-associated SARS-CoV-2 transmission—particularly if conditions that have led to past infections in HCWs with other airborne transmissible diseases, such as measles, remain unchanged.^8–10^ From February 12 to April 9, 2020, a total of 9,282 US HCWs were infected with SARS-Cov-2.^11^ Therefore, we conducted a cross-sectional survey to assess preparedness for COVID-19 cases among hospitals in Idaho.

## Methods

### Survey setting

**Fig. 1.**
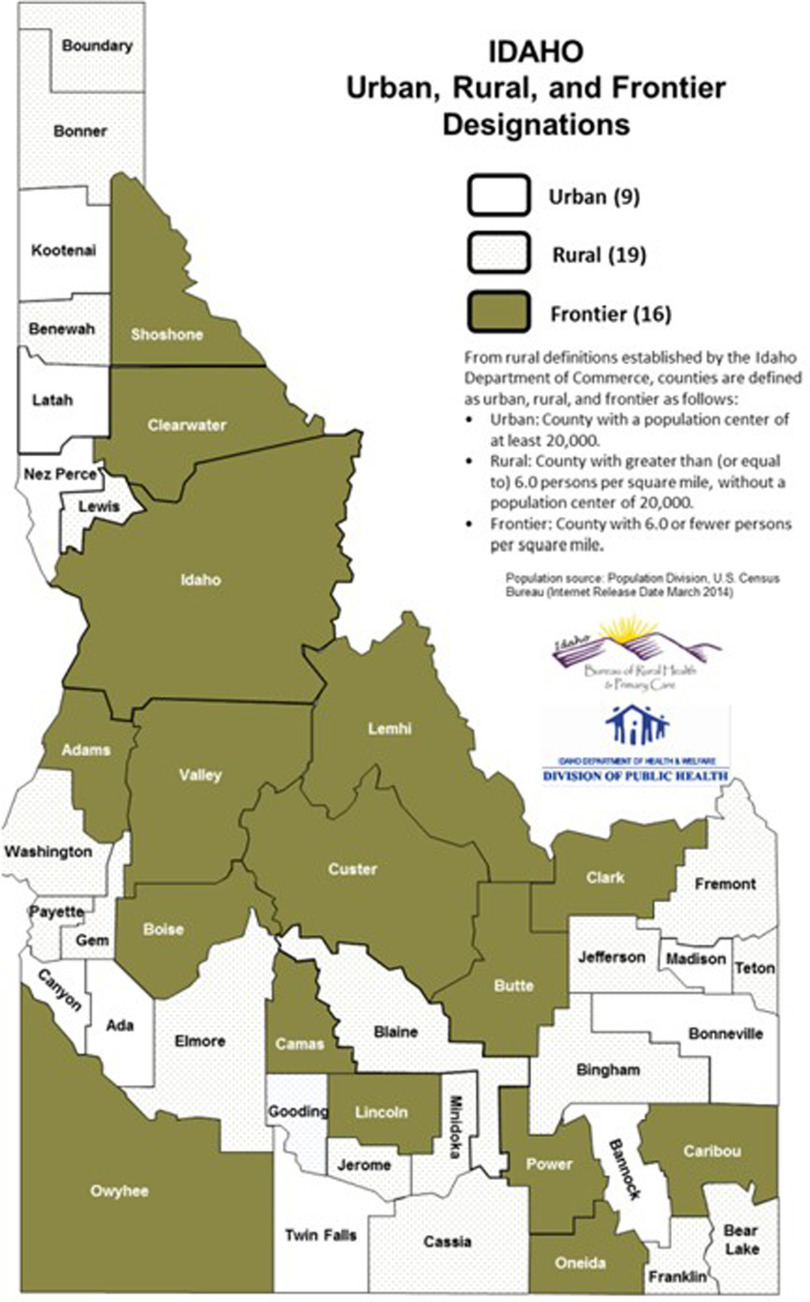
Population density in Idaho.

Idaho has a total population of 1.78 million across its 44 counties.^12^ Figure 1 shows the distribution of population density in frontier (<20,000 population with 6 or fewer people per square mile), rural (<20,000 population but with 6 or >6 people per square mile), and urban (>20,000 population) areas.^13^ Although not all counties have hospitals, each health district has at least 1 hospital, and hospitals serve, on average, 40,450 people.

### Survey design

A 33-item questionnaire was developed to assess institutional policies and practices regarding SARS-CoV-2 detection, management, and infection prevention in acute-care and critical-access hospitals in Idaho. The questions were developed by an infectious disease physician (A.K.), an Idaho public health expert (S.H.), and an occupational and infectious disease epidemiologist (C.B.). The survey focused on organizational structure, availability of facilities and supplies for managing suspected COVID-19 cases, and policies for ensuring protection of HCWs against exposure and infection.

The survey included questions covering the following categories:

- Facility demographics: type of healthcare facility, bed size, zip code, state
- Existing employee health and infection prevention programs, incident command system
- Screening protocols for patients with suspected respiratory illness and travel to countries affected by SARS-CoV-2
- Infrastructure to care for patients with suspected COVID-19 (ie, negative-pressure rooms, availability of N95 masks etc.)
- Respiratory protection program elements

The questionnaire was approved by the St Alphonsus Hospital Institutional Review Board, which granted an exemption because the study did not meet the definition of human subject research.

### Survey distribution

To ensure widespread distribution of the survey, Association of Professionals in Infection Control (APIC) members were contacted through regional chapters in Idaho. APIC membership is the largest network of infection preventionists in the United States. Members include infection preventionists and chief nursing officers (CNOs) working in all 44 hospitals of Idaho: 27 were critical access hospitals (CAHs) and 17 were acute-care hospitals (ACHs). The survey was e-mailed to the infection preventionists and CNOs using an online survey tool (SurveyMonkey, Palo Alto, CA, www.surveymonkey.com). The survey was initially sent on February 6, 2020, with a response deadline of February 23, 2020. An e-mail reminder was sent on February 17, 2020. Survey responses were analyzed using descriptive statistics.

## Results

**Fig. 2.**
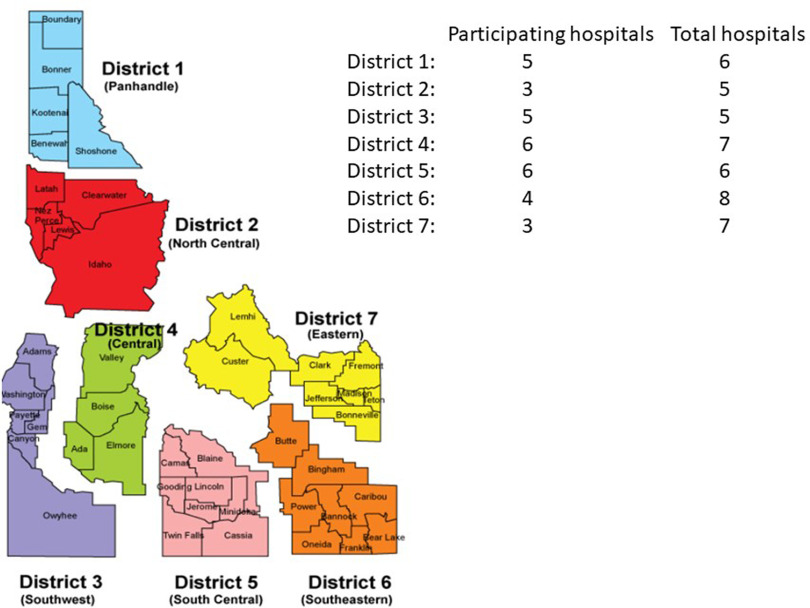
Participating hospitals and their respective regions in Idaho.

In total, 32 (73%) hospitals responded to the survey, with 31 infection preventionists (97%) and 1 CNO (3%) responding on behalf of their respective facilities. Of the responding hospitals, 21 (66%) were CAHs and the remaining 34% were ACHs, which included 5 private and not-for-profit community hospitals (16%), 4 for-profit community hospitals (13%), a state government-owned community hospital, and a Veterans Affairs’ medical center. In addition, 2 hospitals (6%) had ≤10 beds; 20 hospitals (63%) had 11–25 beds; 3 hospitals (9%) had 26–100 beds; 5 hospitals (16%) had 101–200; and 2 hospitals (6%) had >200 beds. The CAHs had <5 intensive care unit (ICU) beds, and the ACHs had >5 ICU beds. The hospitals were from all 7 public health districts within Idaho (Fig. 2). Furthermore, 9 (28%) hospitals were accredited by DNV-GL Healthcare; 12 (38%) were accredited by the Joint Commission; and the remaining 11 hospitals were not accredited. Tables 1–4 list the key findings from the survey. Overall, most (31, 97%) hospitals were aware of CDC interim guidance for healthcare professionals for COVID-19. Also, 14 CAHs (67%) and all 11 ACHs (100%) felt prepared to manage suspected or known COVID-19 cases at their respective hospitals. Moreover, 91% of ACHs reported that they could isolate and manage ~3 patients (range, 1–7) with suspected or known COVID-19 at same time in their hospital, and 81% of CAHs reported that they could isolate and manage ~2 such patients at the same time (range, 0–4). Also, 8 (25%) hospitals, which included 6 (29%) CAHs and 2 (18%) ACHs, reported doing drills to assess preparedness for managing potential COVID-19 cases at their hospitals.

**Table 1.** Elements of Organizational Structure for Supporting Response

Preparedness Element	CAHs (N=21), No. (%)	ACHs (N=11), No. (%)	All Hospitals (N=32), No. (%)
Hospital has an infection control program	21 (100)	11 (100)	32 (100)
Hospital has an incident command system	21 (100)	11 (100)	32 (100)
Hospital has an employee (or occupational) health service	21 (100)	11 (100)	32 (100)

Note. CAH, critical access hospital; ACH, acute-care hospital.

**Table 2.** Ability (ie, policies, supplies, facilities and other resources) to manage a suspected SARS-CoV-2 case

Preparedness Element	CAHs n (%), (N=21)	ACHs n (%), (N=11)	All Hospitals n (%), (N=32)
Has your institution developed any definition for identifying a person under investigation (PUI) for COVID-19/SARS-CoV-2 infection?(Yes)	18 (88)	10 (91)	28 (88)
What precautions are you planning to implement if a COVID-19 PUI comes to your facility?			
Airborne precautions alone	2 (10)	1 (9)	3 (9)
Airborne, contact precautions	6 (29)	2 (18)	8 (25)
Airborne precautions, eye protection devices^a^	1 (5)	0	1 (3)
Airborne and contact precautions, eye protection devices^a^	3 (14)	3 (27)	6 (19)
Airborne, droplet, and contact, precautions, eye protection devices^a^	8 (38)	5 (45)	13 (41)
Droplet precautions alone	1 (5)	0	1 (3)
Are providers at your hospital obtaining a detailed travel history for patients being evaluated with fever and acute respiratory illness? (Yes)	21 (100)	11 (100)	32 (100)
When scheduling appointments, are any special instructions being provided to patients by your institution?			
– Patients should wear a face mask upon arrival if they have any symptoms of respiratory infection (eg, cough, runny nose, fever)	7 (33)	1 (9)	8 (25)
– Patients and accompanying persons should wear a face mask upon arrival if they have symptoms of any respiratory infection (eg, cough, runny nose, fever)	12 (57)	6 (55)	18 (56)
– No special instructions provided but there are posters indicating wearing a face mask upon arrival if they have any symptoms of respiratory infection (eg, cough, runny nose, fever)	0	1 (9)	1 (3)
– No special instructions	2 (10)	3 (27)	5 (16)
Hospital has negative-pressure isolation rooms (airborne infection isolation rooms, or AIIRs)	17 (81)	11 (100)	28 (88)
Designated room or space for a patient with suspected SARS-CoV-2 infection at your institution?	18 (86)	10 (91)	28 (88)
Eye protection devices^a^ are available upon entry to the patient room or care area in following locations:			
Emergency departments	20 (95)	9 (82)	29 (91)
Employee health offices (n=15 for CAHs, n=26 for all hospitals)	7 (47)	6 (55)	13 (50)
Inpatient units	20 (95)	11 (100)	31 (97)
Primary care and specialty clinics	15 (71)	7 (64)	22 (69)
Urgent care (n=14 for CAHs, n=25 for all hospitals)	8 (57)	7 (64)	15 (60)
Hospital has transport plan (to minimize transmission) for moving patients with suspected SARS-CoV-2 cases from emergency room or clinic to designated areas in the institution	14 (67)	10 (91)	24 (75)
Hospital has pre-arrival precautions for a suspected SARS-CoV-2 case:			
Designated hospital entrance	7 (33)	4 (36)	11 (34)
Availability of face masks near patient entrance	21 (100)	11 (100)	32 (100)
Alcohol-hand sanitizer dispenser near the entrance	21 (100)	11 (100)	32 (100)
Procedure for isolating suspected case prior to or as soon as possible after entry into the hospital	19 (90)	11 (100)	30 (94)
How are you planning to manage visitor access and movement within the hospital?			
– Restrict visitors from entering the room of known or suspected SARS-CoV-2 patients unless essential	17 (81)	10 (91)	27 (84)
– Use video-call applications on cell phones or tablets for visitor interaction with patients	3 (14)	2 (18)	5 (16)
– Maintain a logbook of all visitors who enter patient rooms	4 (19)	3 (27)	7 (22)
– Visitors should not be present during aerosol-generating procedures	9 (43)	8 (73)	17 (53)
– In process of modifying or developing new guidance for visitor access and movement	9 (43)	4 (36)	13 (41)
Visual alerts (eg, signs, posters) about hand hygiene, respiratory hygiene, and cough etiquette in your institution:			
Cafeteria	4 (19)	4 (36)	8 (25)
Elevators (n=16 for CAHs)	6 (38)	5 (45)	11 (41)
Hospital entrance	20 (95)	11 (100)	31 (97)
Emergency room	18 (86)	9 (82)	27 (84)
Urgent care (n=14 for CAHs)	13 (93)	8 (73)	21 (84)
Waiting areas	18 (86)	9 (82)	27 (84)
Does your institution have sufficient stocks of the following personal protective equipment (PPE) on hand to manage the anticipated patient volume associated with the outbreak? (affirmative responses)			
Gowns	19 (90)	11 (100)	30 (94)
N95 masks	14 (67)	10 (91)	24 (75)
Surgical masks for patients/visitors	20 (95)	10 (91)	30 (94)
Face shields	15 (71)	11 (100)	26 (81)
Eye protection devices	13 (62)	10 (91)	23 (72)

Note. CAH, critical access hospital; ACH, acute-care hospital.

a Goggles, a disposable face shield that covers the front and sides of the face.

**Table 3.** Testing for Respiratory Viruses

Preparedness Element	CAHs (N=21) No. (%),	ACHs (N=11), No. (%),	All Hospitals (N=32) No. (%),
Influenza A and B	21 (100)	10 (91)	31 (97)
Coronavirus (HKU1, OC43, 229E, or NL63)	2 (10)	6 (55)	8 (25)
Parainfluenza	10 (48)	8 (73)	18 (56)
Respiratory syncytial virus	20 (95)	9 (82)	29 (91)
Rhinovirus	9 (43)	8 (73)	17 (53)
Metapneumovirus	9 (43)	8 (73)	17 (53)
Adenovirus	9 (43)	8 (73)	17 (53)
We do not test for any respiratory viruses at our hospital	0	1 (9)	1 (3)

Note. CAH, critical access hospital; ACH, acute-care hospital.

**Table 4.** Respiratory Protection Program

Preparedness Element	CAHs n(%), (N=21)	ACHs n(%), (N=11)	All Hospitals n(%), (N=32)
N95 FFRs are available in following locations:			
Emergency departments	19 (90)	9 (82)	28 (88)
Employee health offices (n=15 for CAHs, n=26 for all hospitals)	7 (47)	8 (73)	15 (58)
Inpatient units	20 (95)	11 (100)	31 (97)
Primary care and specialty clinics	13 (62)	7 (64)	20 (63)
Urgent care (n=14 for CAHs, n=25 for all hospitals)	7 (50)	8 (73)	15 (60)
Experiencing problems (eg, delays, shortages) in ordering and obtaining respirators (other than N95 FFRs)	11 (52)	2 (18)	13 (41)
Experiencing problems (eg, delays, shortages) in ordering and obtaining N95 FFRs	8 (38)	2 (18)	10 (31)
Written protocol for respiratory protection program (RPP)	19 (90)	10 (91)	29 (91)
Which groups of healthcare workers are fit-tested annually?			
Physicians and providers	17 (81)	9 (82)	26 (81)
Nurses	17 (81)	10 (91)	27 (84)
Nurse-aids	16 (76)	9 (82)	25 (78)
Respiratory therapists	14 (67)	7 (64)	21 (66)
Physical therapists	7 (33)	6 (55)	13 (41)
Occupational therapists	3 (14)	4 (36)	7 (22)
Administrative staff	3 (14)	1 (9)	4 (13)
Environmental cleaning staff	17 (81)	9 (82)	26 (81)
Dietitian	1 (5)	3 (27)	4 (13)
Engineering staff	5 (24)	4 (36)	9 (28)
Security	2 (10)	2 (18)	4 (13)
Laundry	2 (10)	0	2 (6)
Volunteers	0	1 (9)	1 (3)
Contractors	1 (5)	1 (9)	2 (6)
No fit-testing as we use PAPR	0	2 (18)	2 (6)
Type of respiratory protection devices your hospital provides as part of the RPP			
Surgical masks	12 (57)	9 (82)	21 (66)
N95 FFRs	17 (81)	10 (91)	27 (84)
Half-face elastomeric respirators with N95 cartridges	1 (5)	0	1 (3)
Full-face elastomeric respirators with N99 cartridges	0	1 (9)	1 (3)
Full-face elastomeric respirators with N100 cartridges	1 (5)	0	1 (3)
Powered-air purifying respirators with HEPA (or equivalent) cartridges	0	10 (91)	10 (31)
Frequency of respirator training for those covered by RPP at your hospital			
Annually	7 (33)	5 (45)	12 (38)
Upon hire only and as job duties change	2 (10)	1 (9)	3 (9)
Upon hire, then annually	4 (19)	1 (9)	5 (16)
Annually and as job duties change	0	3 (27)	3 (9)
Upon hire, then annually and as job duties change	7 (33)	1 (9)	8 (25)
Do not provide any respirator training	1 (5)	0	1 (3)

Note. CAH, critical access hospital; ACH, acute-care hospital; PAPR, powered, air-purifying respirator; FFR, filtering face-piece respirator; HEPA, high-efficiency particulate air filter.

## Discussion

In light of recently recognized community transmission of SARS-CoV-2 in the United States (where the most cases and deaths have occurred thus far), this study highlights critical components of COVID-19 preparedness among hospitals, including CAHs and ACHs.

All responding hospitals had the basic organizational structure for facility-wide prevention and management efforts in case a patient with suspected or confirmed COVID-19 presented to that facility. Most of the hospitals also had at least some available resources to manage COVID-19 cases, including measures for protecting HCWs. In this section, we further examine some of these preparedness successes and potential gaps.

All responding hospitals reported organizational structures that support facility-level response to infectious disease events. These include the across-the-board implementation of defined infection control programs, incident command structures, and employee and occupational health services within all hospitals.

Despite reported shortages of PPE elsewhere in the country, the surveyed hospitals generally reported having sufficient quantities of gowns, face shields, surgical masks, and respirators for managing their anticipated patient load during an outbreak scenario. Of all types of PPE, hospitals least frequently reported having eye protection equipment (other than face shields, which would serve a similar purpose) on hand. This likely indicates a preference for face shields over goggles among the responding hospitals and not a shortage of eye protection. However, because face shields are disposable, they may be more prone to supply shortages than goggles, which can be decontaminated and reused.

All CAHs that responded to the question about the availability of N95 respirators in the facility indicated that such devices are available in at least 1 location—although not all locations—where COVID-19 cases are likely to present for care. Nearly all facilities reported keeping N95s in inpatient units and emergency departments; however, the survey also revealed some instances in which <50% of the other hospital-affiliated locations had N95s, such as urgent care clinics where patients with COVID-19 might present. Notably, given current federal guidance for optimizing supplies of respiratory protective devices in the healthcare sector, responding hospitals reported, in at least a few instances, using respirator types other than N95 filtering face-piece respirators (FFRs) in their respiratory protection programs. These included elastomeric air-purifying respirators with various types of cartridges, as well as powered, air-purifying respirators (PAPRs).

Consistent with widely accepted best practices and lessons learned from previous outbreaks, all hospitals reported at least some measures aimed at promoting early identification and isolation of potentially infectious patients. Specifically, all responding hospitals reported that staff are trained to collect a detailed travel history for patients with signs or symptoms of respiratory illnesses, including COVID-19. More than half of facilities reported requesting that arriving patients, or patients and their accompanying parties, should wear face masks to contain potentially infectious respiratory sections. All facilities indicated that they provide masks near the patient entrance. Most hospitals also reported posting signage about respiratory and/or hand hygiene in 1 or more locations around the facility.

Most facilities indicated that they have a dedicated room or space for suspected COVID-19 patients, and most also reported having a plan to move patients from intake areas, such as the emergency department, to that place. However, other isolation facilities were scarce. Fewer than half of hospitals that responded to the survey mentioned instructing potentially infectious patients to use a dedicated entrance to the facility, and even fewer had airborne infection isolation rooms (AIIRs) in which to isolate potentially infectious patients after arrival and triage.

The survey also revealed several other areas in which preparedness for COVID-19 cases could be improved. For example, hospitals did not report uniformly following the CDC’s recommended transmission-based precautions, which include contact and airborne precautions with additional face and eye protection (eg, goggles or face shields), when HCWs interact with potentially infectious COVID-19 patients. The largest group of facilities reported using a combination of contact, droplet, and airborne precautions plus eye protection, which may indicate that hospitals are following CDC guidelines. However, it was not immediately clear how, when, or why personnel would switch between droplet and airborne precautions. This lack of clarity was likely a limitation of the survey. The second most common response to this survey item indicated that hospitals were following contact and airborne precautions, potentially omitting eye protection necessary to protect HCWs under the prevailing CDC guidelines.

Among the potentially more serious gaps is the finding that not all hospitals reported maintaining written respiratory protection programs. Where respirators are required to protect workers from infectious materials in the air, including SARS-CoV-2, the Occupational Safety and Health Administration (OSHA) requires such programs under its respiratory protection standard.^14^ Having a written program also supports the implementation of requirements for fit testing, training, medical exams, and election and use of National Institute for Occupational Safety and Health (NIOSH)–certified respirators appropriate to protect workers from respiratory hazards in the workplace. Not all hospitals reported complying with OSHA requirements for initial and annual fit testing. It is unclear whether this deviation from what would be expected under full compliance is due to hospital concerns over respirator supply usage associated with annual fit testing (ie, facilities may have decided on their own to temporarily bypass this requirement in an attempt to conserve respirators) or if an incomplete fit-testing protocol is the norm in such facilities. Despite facing possible supply shortages associated with the SARS-CoV-2 outbreak, maintaining, at a minimum, the other elements of required respiratory protection programs can help to ensure continued worker protection against exposure to SARS-CoV-2.

Although our survey provides an initial perspective on COVID-19 preparedness among Idaho hospitals, it is important to acknowledge that it was limited to 1 state and did not include other healthcare facilities, such as nursing homes, that have been associated with the ongoing SARS-CoV-2 outbreak. The study was also cross-sectional in design, relying on self-reported survey data from hospital infection preventionists. We also did not examine SARS-CoV-2 testing by hospitals because it was only done through the Idaho State Public Health Laboratory and the CDC at the time of survey. COVID-19 infection prevention and control policies and practices may vary significantly among different types of facilities and/or those in different states. However, all hospitals should be following CDC guidance, particularly until more is known about SARS-CoV-2 transmission patterns and the risks associated with various exposure routes and scenarios.

Future research focused on differences in outbreak readiness and capabilities among various facilities may help identify factors influencing preparedness. Such studies could further examine available intensive care unit beds, PPE burn rates and stockpile requirements, as well as the capacity to handle a surge in COVID-19 cases among both smaller and larger facilities.

Despite these limitations, this study may be useful to hospitals throughout the Pacific Northwest region, or other types of healthcare facilities, particularly because it highlights areas where they may wish to examine their own levels of preparedness. As the ongoing pandemic has highlighted, pathogens do not respect borders and most, if not all, states face preparedness challenges of varying degrees. Because the survey covers so many aspects of preparedness for managing respiratory viruses that may cause serious and/or widespread outbreaks, this study also offers a model for shaping future explorations of pandemic preparedness among hospitals.
